# Synchrotron X-ray fluorescence studies of a bromine-labelled cyclic RGD peptide interacting with individual tumor cells

**DOI:** 10.1107/S0909049513001647

**Published:** 2013-02-07

**Authors:** Erin J. Sheridan, Christopher J. D. Austin, Jade B. Aitken, Stefan Vogt, Katrina A. Jolliffe, Hugh H. Harris, Louis M. Rendina

**Affiliations:** a School of Chemistry, The University of Sydney, Sydney, NSW 2006, Australia; b Australian Synchrotron, Clayton, Victoria 3168, Australia; c Institute of Materials Structure Science, KEK, Tsukuba, Ibaraki 305-0801, Japan; d X-ray Science Division, Argonne National Laboratory, Argonne, IL 60439, USA; e School of Chemistry and Physics, The University of Adelaide, Adelaide, South Australia 5005, Australia

**Keywords:** XRF, microprobe, RGD peptide, tumor cells

## Abstract

The first example of synchrotron X-ray fluorescence imaging of cultured mammalian cells in cyclic peptide research is reported. The study reports the first quantitative analysis of the incorporation of a bromine-labelled cyclic RGD peptide and its effects on the biodistribution of endogenous elements (for example, K and Cl) within individual tumor cells.

## 1. Introduction   

More than a century ago, an association was made between the growth of a tumor and the circulatory connections made to existing host vasculature (Goldmann, 1908 ▶). A tumor, like all tissues, requires access to essential nutrients and oxygen carried in the blood for continued survival (Vaupel *et al.*, 1989 ▶). A subsequent century of research has found that malignancies can co-opt key cellular mechanisms to form new vascular links to existing host blood vessels (*i.e.* angiogenesis) and create new blood vessels within the metastasis (*i.e.* neovascularization) (Bouck, 1990 ▶; Folkman, 1985 ▶, 1995 ▶; Plate *et al.*, 1992 ▶).

As tumor-induced angiogenesis proceeds, a number of activated oncogenic molecules (*e.g.* vascular endothelial growth factor, angiopoietin-1, and basic fibroblast growth factor) stimulate endothelial cell proliferation, migration and assembly to initiate new blood vessel growth (Veikkola *et al.*, 2000 ▶; Koblizek *et al.*, 1998 ▶; Montesano *et al.*, 1986 ▶). This process is mediated by extracellular matrix (ECM) receptor integrins, which anchor growing cells in the ECM and control signalling and organization of the intracellular actin cyto­skeleton (Eliceiri & Cheresh, 1999 ▶; Stupack & Cheresh, 2004 ▶).

**Figure 1:**
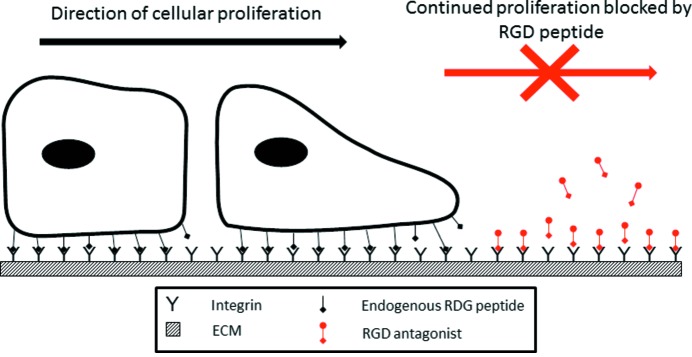
Cell proliferation and angiogenesis requires interaction between the cell and the extracellular matrix that is mediated by integrins. Endogenous RGD peptides bind to integrins and anchor migrating and dividing cells. The desired mode of action of RGD antagonists is to block endogenous RGD peptide binding, thus preventing continued cellular proliferation and angiogenesis.

The Arg-Gly-Asp (RGD) sequence is a key recognition motif for integrin receptors (Ruoslahti & Pierschbacher, 1987 ▶; Ruoslahti, 1996 ▶), and the conformation of the RGD-containing loop and the flanking amino acids are predominantly responsible for the binding affinity of RGD-containing cell surface peptides to integrin molecules (Ruoslahti & Pierschbacher, 1987 ▶; Ruoslahti, 1996 ▶). Consequently, interest in integrin targeting for anti-angiogenic therapies has escalated, as the binding of antagonistic RGD-receptor ligands has been shown to inhibit processes such as tumor-induced angiogenesis (Dijkgraaf *et al.*, 2007 ▶). Interestingly, the expression of a particular integrin, α_v_β_3_, has been shown to correlate directly with tumor grade and thus represents an attractive molecular target to prevent tumor proliferation and spread (Fig. 1 ▶) (Dijkgraaf *et al.*, 2007 ▶). Indeed, cilengitide, an *N*-alkylated cyclic peptide c(RGDf[NMe]V) α_v_ integrin antagonist which disrupts angiogenesis (Avraamides *et al.*, 2008 ▶; Herbst, 2006 ▶; Jain *et al.*, 2007 ▶), is currently in Phase III clinical trials for the treatment of glioblastoma multiforme (Reardon *et al.*, 2008 ▶).

Despite the many examples of successfully conjugating mono-, di- and multi-meric cyclic RGD peptides to a variety of imaging agents (Chen *et al.*, 2004*a* ▶,*b* ▶; Kaim & Schwederski, 1994 ▶), including radiolabels such as ^18^F, ^64^Cu, ^68^Ga, ^76^Br and ^89^Zr for PET imaging (Chen *et al.*, 2004*c* ▶,*d* ▶; Li *et al.*, 2008 ▶; Lang *et al.*, 2011 ▶; Temming *et al.*, 2006 ▶), ^99*m*^Tc, ^111^In and ^125^I for SPECT imaging (Jia *et al.*, 2006 ▶; Edwards *et al.*, 2009 ▶; Haubner *et al.*, 2001 ▶), Gd nanotubes for MRI (Mulder *et al.*, 2005 ▶), and near-IR dyes such as cypate (Edwards *et al.*, 2009 ▶) for *in vivo* optical imaging, few studies offer high-resolution images of the uptake of RGD peptides within a *single* tumor cell. Synchrotron X-ray fluorescence (XRF) is a powerful microanalytical tool which offers multi-element imaging at sub­micrometre resolution for those elements with *Z* > 11 (Crossley *et al.*, 2011 ▶). XRF can identify changes in elemental content and localization within biological samples, including whole cells (Dillon *et al.*, 2002 ▶; Harris *et al.*, 2005 ▶, 2008 ▶), and it has been successfully used to determine the elemental biodistribution within tumor cells treated with Pt(II) and Gd(III) complexes (Crossley *et al.*, 2010 ▶, 2011 ▶; Hall *et al.*, 2003 ▶).

As RGD antagonist peptides typically contain only light elements (*i.e.* H, C, N and O), their detection is difficult due to poor sensitivity at their low XRF energies and, more significantly, a very high background within cells for these endogeneous elements. Therefore, conjugation of a heavier element, such as a halogen, at a position remote to the substrate binding site by using a method akin to radiolabelling (*e.g.* ^18^F or ^125^I) is a viable option for XRF imaging. Bromine is an ideal candidate for XRF labelling as its *K*α_1_ transition is accessible using XRF techniques and C—Br bonds are quite robust within biological systems. Significantly, bromine occurs naturally in the body at very low levels (∼3–4 p.p.m. in blood serum) (Lyon *et al.*, 2005 ▶; Underwood, 1971 ▶) and is generally non-toxic when bound to organic molecules. Bromine has been used previously as a heavy-atom label for X-ray crystallographic studies involving parasymphatolytic drug production and DNA labelling (Paradellis & Skouras, 1979 ▶; Zeitz & Lee, 1963 ▶).

Herein we report the synthesis and cell binding of a bromo-labelled cyclic RGD peptide, **1** [Chem scheme1]. We also report the effect of RGD peptide binding on endogenous elements such as K and Cl and demonstrate that the synchrotron XRF technique is a viable method for imaging individual cells when a heavy-atom label is incorporated into an important class of synthetic cyclic peptides.

## 2. Experimental   

Unless otherwise specified, all reactions were carried out under an inert atmosphere of dry nitrogen using conventional Schlenk techniques (Shriver, 1969 ▶), using dry freshly distilled solvents. All chemicals and reagents were purchased from Aldrich, Auspep, Boron Molecular, Fluka or Novabiochem and used without further purification. Dimethylsulfoxide (DMSO) and peptide-grade *N*,*N*-dimethylformamide (DMF) were used without further distillation. Dichloromethane (DCM) was dried over calcium hydride. Analytical and preparative reverse-phase HPLC (RP HPLC) was performed using a Waters apparatus with a photodiode array absorbance detector. Analytical RP HPLC was performed on an Alltech-Altima C18 column (5 µm, 4.6 mm ID, 250 mm) with a 0.8 ml min^−1^ flow rate. Preparative RP HPLC was performed using an Alltech-Altima C18 column (10 µm, 22 mm ID, 300 mm) with a 7 ml min^−1^ flow rate. Melting points were obtained using a Stuart SMPII melting-point apparatus with a mercury thermometer and microscope, and are uncorrected.

Optical rotations were recorded on a POLAAR 2001 polarimeter at 546 nm with a cell path of 0.25 or 0.50 dm. NMR spectra were obtained on a 300 MHz Bruker spectrometer at 300 ± 1 K. ^1^H NMR spectra were recorded at 300 MHz and ^13^C at 75 MHz. ^1^H and ^13^C NMR spectra were referenced to residual solvent peaks (δ_H_ 3.31 and δ_C_ 49.0 for CD_3_OD). All NMR spectra were recorded in p.p.m., with coupling constants reported in Hz. Low-resolution ESI-MS data were acquired using a Finnigan LCQ detector. High-resolution ESI-MS data were acquired using either a Bruker 7.0 T or a Bruker Apex 4.7 T spectrometer.

### 2.1. H_2_N-Asp(O^*t*^Bu)-d-Phe(4-Br)-Val-Arg(Pbf)-Gly-OH (**2**)   

#### 2.1.1. Loading of 2-chlorotrityl resin   

A solution of Fmoc-Gly-OH (3.91 g, 13 mmol) and Hünigs base (3.7 ml, 21 mmol) in DCM:DMF (6:1, 35 ml) was added to 2-chlorotrityl chloride resin and the reaction mixture was agitated for 2 h. After this time the solution was removed by filtration, and a solution of MeOH (10 ml, 0.25 mol) and Hünigs base (2.5 ml, 14 mmol) was added to cap any unreacted sites. After shaking the mixture for a further 45 min the resin was washed with DMF (4 × 20 ml), DCM (2 × 20 ml), MeOH (2 × 20 ml), diethyl ether (2 × 20 ml), and the resulting resin dried *in vacuo* and stored under nitrogen. The loading of the resin was calculated to be 1.65 mmol g^−1^ of resin.

#### 2.1.2. Peptide synthesis   

The synthesis was carried out using standard Fmoc protocols on a PS3 automated solid phase peptide synthesiser. The Fmoc-protected amino acids (2 equivalents relative to resin loading) and HBTU (2 equivalents relative to resin loading) were packed dry into reaction vials. Hünigs base (0.4 *M* in DMF) was used as the activating solution for the coupling reactions; and piperidine (20% *v*/*v* in DMF) was used for Fmoc-deprotection.

#### 2.1.3. Cleavage   

The resin was treated with a solution of acetic acid, TFE and DCM (1:1:3, 20 ml) for 2 h. The solution was drained and the resin washed with a solution of acetic acid and DCM (1:1, 2 × 50 ml), DCM (2 × 50 ml) and then DMF (2 × 50 ml). The organic extracts were combined and the solvent was removed *in vacuo* to give the required pentapeptide **2** as a colourless solid. The peptide was found to be pure by analytical RP HPLC (480 mg, 98%). M.p. 493–498 K. [α]_D_ = +21.3 (*c*. 0.4, CHCl_3_). ^1^H NMR (300 MHz, CD_3_OD): δ 7.48 (*d*, 2H, ^3^ *J*_HH_ = 8.1), 7.26 (*d*, 2H, ^3^ *J*_HH_ = 7.9), 5.50 (*s*, 2H), 4.77 (*t*, 1H, ^3^ *J*_HH_ = 7.8), 4.43 (*q*, 1H, ^3^ *J*_HH_ = 4.8), 4.36 (*s*, 2H), 4.16 (*t*, 1H, ^3^ *J*_HH_ = 6.0), 3.95 (*d*, 2H, ^3^ *J*_HH_ = 15.9), 3.86 (*s*, 1H), 3.20 (*t*, 2H, ^3^ *J*_HH_ = 6.0), 2.55 (*d*, 2H, ^3^ *J*_HH_ = 17.1), 2.36 (*s*, 6H), 2.11 (*s*, 3H), 1.79 (*s*, 2H), 1.56–1.54 (*m*, 2H), 1.49 (*s*, 9H), 1.47 (*s*, 6H), 1.31 (*t*, 2H, ^3^ *J*_HH_ = 6.0), 0.81 (*d*, 6H, ^3^ *J*_HH_ = 6.9), NH_3_^+^, NH and OH not observed. ^13^C{1H} NMR (75 MHz, CD_3_OD): δ 176.3, 175.9, 173.7, 172.9, 171.8, 161.3, 155.5, 135.0, 134.3, 133.7, 133.4, 133.1, 132.6, 128.9, 128.3, 127.8, 83.6, 80.7, 69.2, 67.2, 57.2, 56.4, 56.1, 54.3, 46.1, 44.5, 37.5, 32.2, 29.9, 29.1, 24.4, 23.8, 19.2, 18.2. ESI-MS *m*/*z* 979.6, 981.6 ([*M* + H]^+^, 100%). High-resolution ESI-MS *m*/*z* calculated for [*M* + Na]^+^, 1001.3418, 1003.3398; found, 1001.3421, 1003.3400. RP HPLC retention time 23.4 min.

### 2.2. Cyclo[Gly Arg(Pbf)-Val-d-(4-Br)Phe-Asp(O^*t*^Bu)] (**3**)   

The linear fully protected peptide **2** (234 mg, 0.23 mmol) was dissolved in DMF (final peptide concentration = 1 × 10^−4^ *M*), and treated with FDPP (2.5 equivalents) and Hünigs base (4 equivalents). The solution was stirred for 96 h at room temperature. The solvent was removed *in vacuo* and the residue dissolved in ethyl acetate (50 ml), washed with 1 *M* HCl (2 × 40 ml), aqueous saturated NaHCO_3_ (2 × 40 ml) and brine (2 × 40 ml), then dried over Na_2_SO_4_. The solvent was removed *in vacuo* and the residue purified by preparative RP HPLC to afford **3** as a colourless solid (113 mg, 49%). M.p. 401–408 K. [α]_D_ = +8.52 (*c*. 0.1, CHCl_3_). ^1^H NMR (300 MHz, CD_3_OD): δ 7.46 (*d*, 2H, ^3^ *J*_HH_ = 7.6), 7.22 (*d*, 2H, ^3^ *J*_HH_ = 8.0), 5.65 (*s*, 2H), 4.72 (*t*, 1H, ^3^ *J*_HH_ = 7.6), 4.40 (*q*, 1H, ^3^ *J*_HH_ = 5.2), 4.35 (*s*, 2H), 4.11 (*t*, 1H, ^3^ *J*_HH_ = 6.6), 3.99 (*d*, 2H, ^3^ *J*_HH_ = 12.8), 3.82 (*s*, 1H), 3.20 (*t*, 2H, ^3^ *J*_HH_ = 5.8), 2.42 (*d*, 2H, ^3^ *J*_HH_ = 14.3), 2.33 (*s*, 6H), 2.16 (*s*, 3H), 1.77 (*s*, 2H), 1.55–1.51 (*m*, 2H), 1.49 (*s*, 9H), 1.43 (*s*, 6H), 1.37 (*t*, 2H, ^3^ *J*_HH_ = 6.6), 0.86 (*d*, 6H, ^3^ *J*_HH_ = 6.3), NH not observed. ^13^C{^1^H} NMR (75 MHz, CD_3_OD): δ 175.7, 173.2, 172.6, 172.4, 170.5, 160.3, 155.2, 135.2, 134.8, 134.2, 133.5, 133.0, 132.4, 128.3, 127.5, 127.2, 84.5, 81.0, 69.9, 68.7, 57.9, 56.8, 56.3, 54.7, 47.2, 46.1, 44.4, 37.8, 32.4, 29.9, 29.7, 24.7, 24.0, 19.6, 18.3. ESI-MS *m*/*z* 961.8, 963.9 ([*M* + H]^+^, 100%). High-resolution ESI-MS *m*/*z* calculated for [*M* + H]^+^, 961.3493, 963.3473; found, 961.3489, 963.3470. RP HPLC retention time 29.2 min.

### 2.3. Cyclo[Gly Arg-Val-d-(4-Br)Phe-Asp] (**1**)   

The fully protected cyclic peptide **3** (74 mg, 0.077 mmol) was dissolved in a mixture containing TFA:phenol:ethanedithiol (95:4:1, 20 ml) and it was stirred at room temperature for 2 h. The solvent was removed *in vacuo* and the residue taken up into CHCl_3_:*i*-propanol (3:1, 20 ml). The solution was washed with saturated NaHCO_3_ (2 × 15 ml) and brine (2 × 15 ml), dried over Na_2_SO_4_ and the solvent removed *in vacuo*. The crude product was purified by RP flash column chromatography to afford **1** as a colourless solid after preparative RP HPLC (36 mg, 72%). M.p. 480–486 K. [α]_D_ = +3.55 (*c*. 0.05, MeOH). ^1^H NMR (300 MHz, CD_3_OD): δ 7.44 (*d*, 2H, ^3^ *J*_HH_ = 6.9), 7.27 (*d*, 2H, ^3^ *J*_HH_ = 7.2), 5.11 (*s*, 2H), 4.67 (*t*, 1H, ^3^ *J*_HH_ = 7.2), 4.44 (*q*, 1H, ^3^ *J*_HH_ = 5.5), 4.08 (*t*, 1H, ^3^ *J*_HH_ = 6.8), 3.94 (*d*, 2H, ^3^ *J*_HH_ = 11.7), 3.88 (*s*, 1H), 3.25 (*t*, 2H, ^3^ *J*_HH_ = 5.2), 2.46 (*d*, 2H, ^3^ *J*_HH_ = 12.9), 1.79 (*s*, 2H), 1.53–1.48 (*m*, 2H), 1.34 (*t*, 2H, ^3^ *J*_HH_ = 7.2), 0.82 (*d*, 6H, ^3^ *J*_HH_ = 6.8), NH and OH not observed. ^13^C{^1^H} NMR (75 MHz, CD_3_OD): δ 176.1, 173.9, 173.3, 172.8, 172.4, 170.8, 161.1, 135.6, 132.3, 127.8, 69.0, 58.2, 56.5, 55.3, 54.8, 46.4, 44.9, 37.7, 32.0, 29.9, 29.5, 24.1, 18.3. ESI-MS *m*/*z* 653.5, 655.8 ([*M* + H]^+^, 100%). High-resolution ESI-MS *m*/*z* calculated for [*M* + H]^+^, 653.2047, 655.2026; found, 653.2053, 655.2032. RP HPLC retention time 24.2 min.

### 2.4. XRF studies of A549 human lung carcinoma and B16 murine melanoma cells   

All cells used for XRF imaging were grown directly on 1.5 × 1.5 mm 500 nm-thick silicon nitride windows (Silson Pty, UK) in six-well plates as described previously (Crossley *et al.*, 2010 ▶). Control A549 human lung carcinoma and B16 murine melanoma cells were incubated separately for 24 h in PBS buffer, while treated cells were incubated for 24 h with **1** (30 µ*M* for A549, 75 µ*M* for B16) in PBS buffer. Following treatment the cells were washed twice in PBS, then fixed on the silicon nitride windows by dipping in methanol (5X). Hard X-ray microprobe imaging experiments were performed on XOR (X-ray Operations Research) beamline 2ID-D for both control and treated A549 cells as well as treated B16 cells. Elemental maps of control B16 cells were collected on beamline 2ID-E; previous work has shown that the same cell produced comparable results on both 2ID-D and 2ID-E (H. H. Harris, J. B. Aitken & S. Vogt, unpublished). On both beamlines a monochromatic 13.87 keV X-ray incident beam using a beam splitting Si(220) monochromator was focused to a diameter of ∼0.5 µm using either a single zone plate (2-ID-E) or dual zone plate set-up (2-ID-D). A single-element silicon drift energy-dispersive detector (Vortex EX, SII Nanotechnology, Northridge, CA, USA), at 90° to the incident beam, was used to collect the fluorescence signal for 1 s per spatial point from samples under a He atmosphere.

### 2.5. Data and statistical analysis   

The fluorescence spectrum collected at each spatial point was fit with modified Gaussians (van Espen, 2002 ▶) and quantification for each element of interest was performed by comparison with the corresponding measurements on the thin-film standards NBS-1832 and NBS-1833 from the National Bureau of Standards (Gaithersburg, MD, USA) using *MAPS* software (Vogt, 2003 ▶). Regions of interest in the XRF images corresponding to a whole cell (identified using optical images and the elemental distribution maps of P, S, Cl, K and Zn) were selected and determination of cell areas (as reported in Table 1 ▶) was based on these regions. The integrated fluorescence spectra extracted from these regions were also quantified, as described above, to determine average elemental area densities (in units of µg cm^−2^) in the cells. The mean elemental densities for a number of cells (*n* = 5) for each treatment are reported in Table 1 ▶. The statistical significance of the difference between mean elemental densities for the treated cells for each cell line against that of the controls was assessed using the independent samples two-tailed Mann Whitney test with a criterion of *p* ≤ 0.05 (Phipps & Quine, 2001 ▶).

## 3. Results   

### 3.1. Synthesis of cyclic RGD peptide **1**   

The required linear precursor NH_2_-Asp(O^*t*^Bu)-d-Phe(4-Br)-Val-Arg(Pbf)-Gly-OH (see scheme[Chem scheme1]) was synthesized by standard solid-phase peptide synthesis techniques using Fmoc/HBTU chemistry on a PS3 synthesiser. The 2-chlorotrityl resin was used as the solid support to enable cleavage of the peptide from the solid phase under mildly acidic conditions [acetic acid:trifluoroethanol:dichloromethane (1:1:3)] with side-chain protecting groups intact to give **2** in very high yield (98%) after RP HPLC. Cyclization of **2** was successfully accomplished using FDPP and Hünigs base as the coupling reagents in DMF at a concentration of 1 × 10^−4^ *M* for 96 h, giving **3** in 49% yield (see scheme[Chem scheme1]). Treatment of **3** with a mixture of TFA, and the nucleophilic scavengers phenol and ethanedithiol (95:4:1) gave the desired cyclic RGD peptide **1** in 72% yield following RP HPLC (see scheme[Chem scheme1]).

### 3.2. XRF elemental density: cells (A549 and B16) treated with **1** and (A549 and B16) control cells   

**Figure 2:**
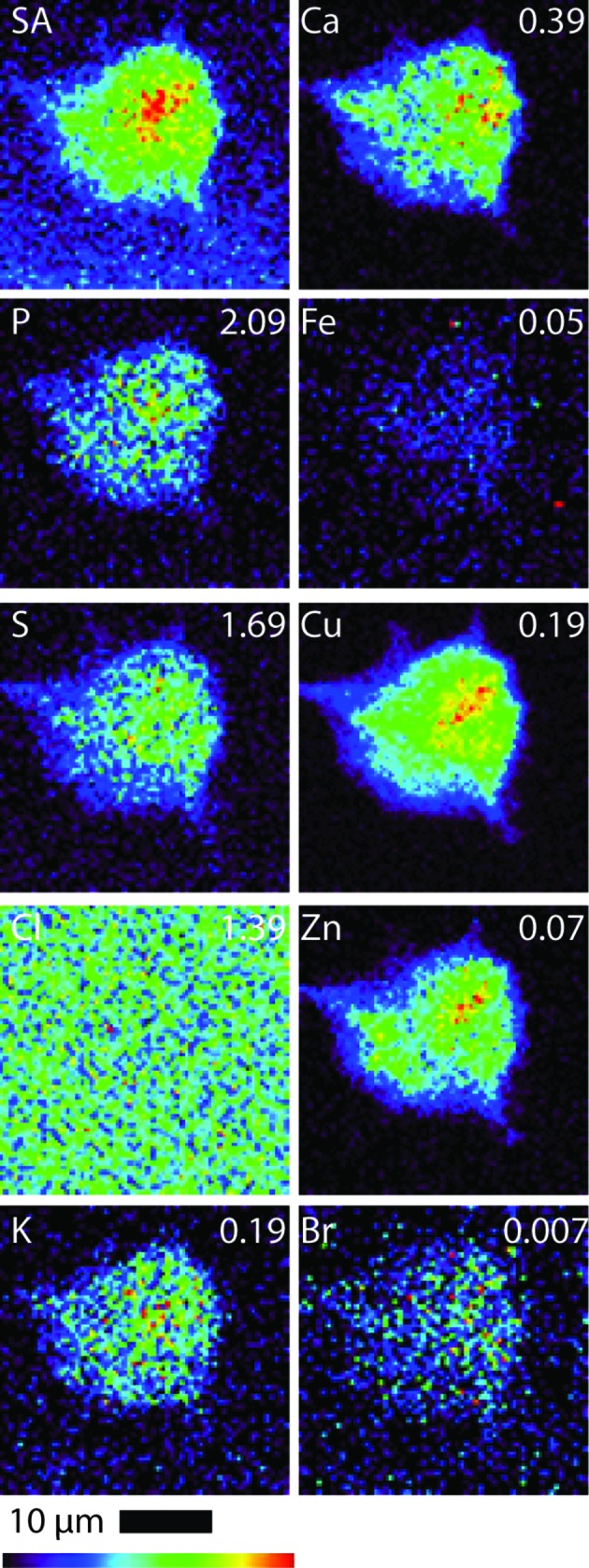
Scattered X-ray (SA) and XRF elemental distribution maps of P, S, Cl, K, Ca, Fe, Cu, Zn and Br of an B16 cell treated with **1** for 24 h. The maximal elemental area density (in µg cm^−2^) is given in the top corner of each map.

**Figure 3:**
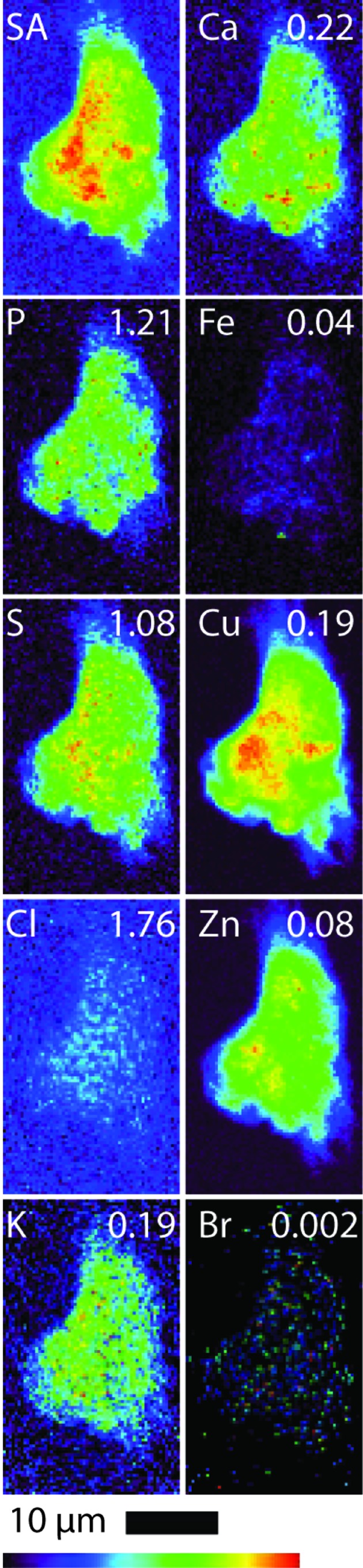
Scattered X-ray (SA) and XRF elemental distribution maps of P, S, Cl, K, Ca, Fe, Cu, Zn and Br of an untreated B16 cell. The maximal elemental area density (in µg cm^−2^) is given in the top corner of each map.

**Figure 4:**
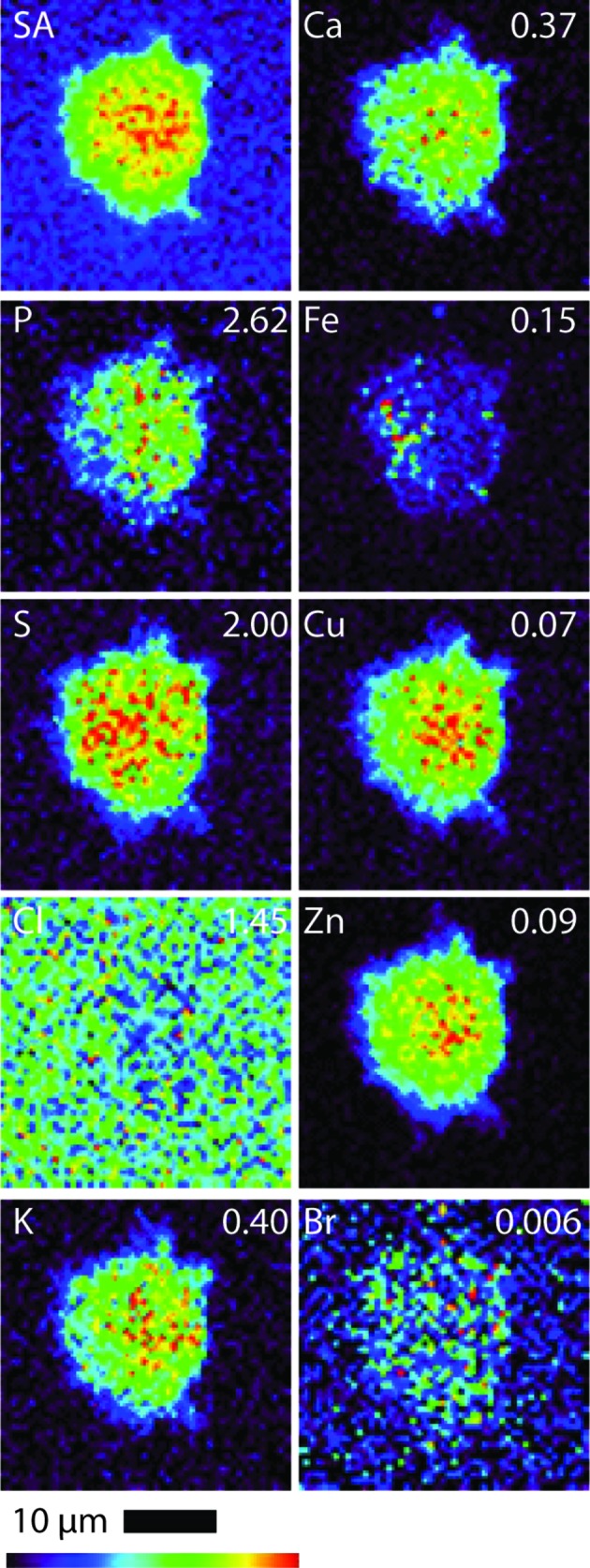
Scattered X-ray (SA) and XRF elemental distribution maps of P, S, Cl, K, Ca, Fe, Cu, Zn and Br of an A549 cell treated with **1** for 24 h. The maximal elemental area density (in µg cm^−2^) is given in the top corner of each map.

**Figure 5:**
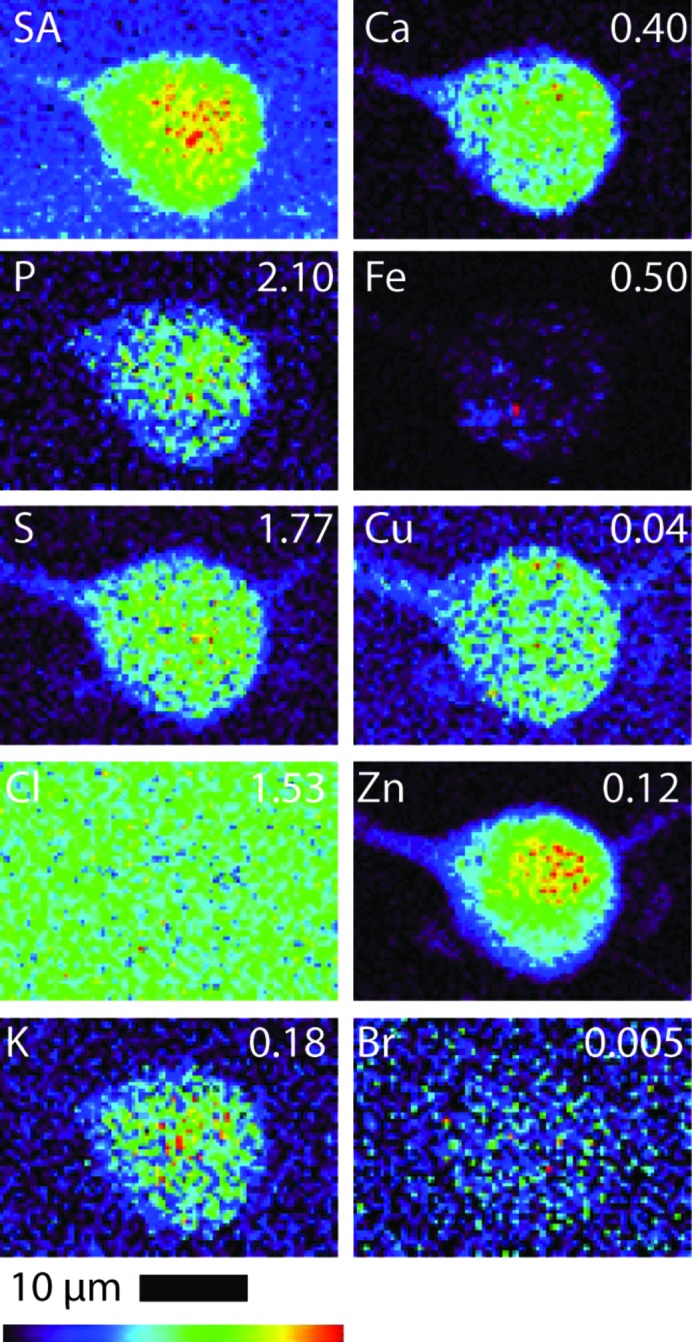
Scattered X-ray (SA) and XRF elemental distribution maps of P, S, Cl, K, Ca, Fe, Cu, Zn and Br of an untreated A549 cell. The maximal elemental area density (in µg cm^−2^) is given in the top corner of each map.

All B16 (murine melanoma) and A549 (adenocarcinomic human alveolar basal epithelial) cells (both treated and control cells) were regular in shape and showed an overlap in Zn and P elemental maps, indicating that the integrity of the cells and cell nucleus was maintained during the fixing and washing procedures (*i.e.* there was no evidence of blebbing) (Crossley *et al.*, 2011 ▶). Table 1 ▶ shows the quantified area and elemental densities of treated A549 and B16 cells, respectively. Figs. 2 and 3 show the X-ray elastic scatter map and elemental maps for typical treated B16 (24 h incubation with 75 µ*M* of **1**) and the appropriate untreated control (B16 control cell A), respectively. Figs. 4 and 5 show the X-ray elastic scatter map and elemental maps for a typical treated A549 cell (24 h incubation with 30 µ*M* of **1**) and the appropriate untreated control (A549 control cell A), respectively. Data on all cells (treated/control: area measurements; X-ray elastic scattering) are presented as supplementary material.^1^ 
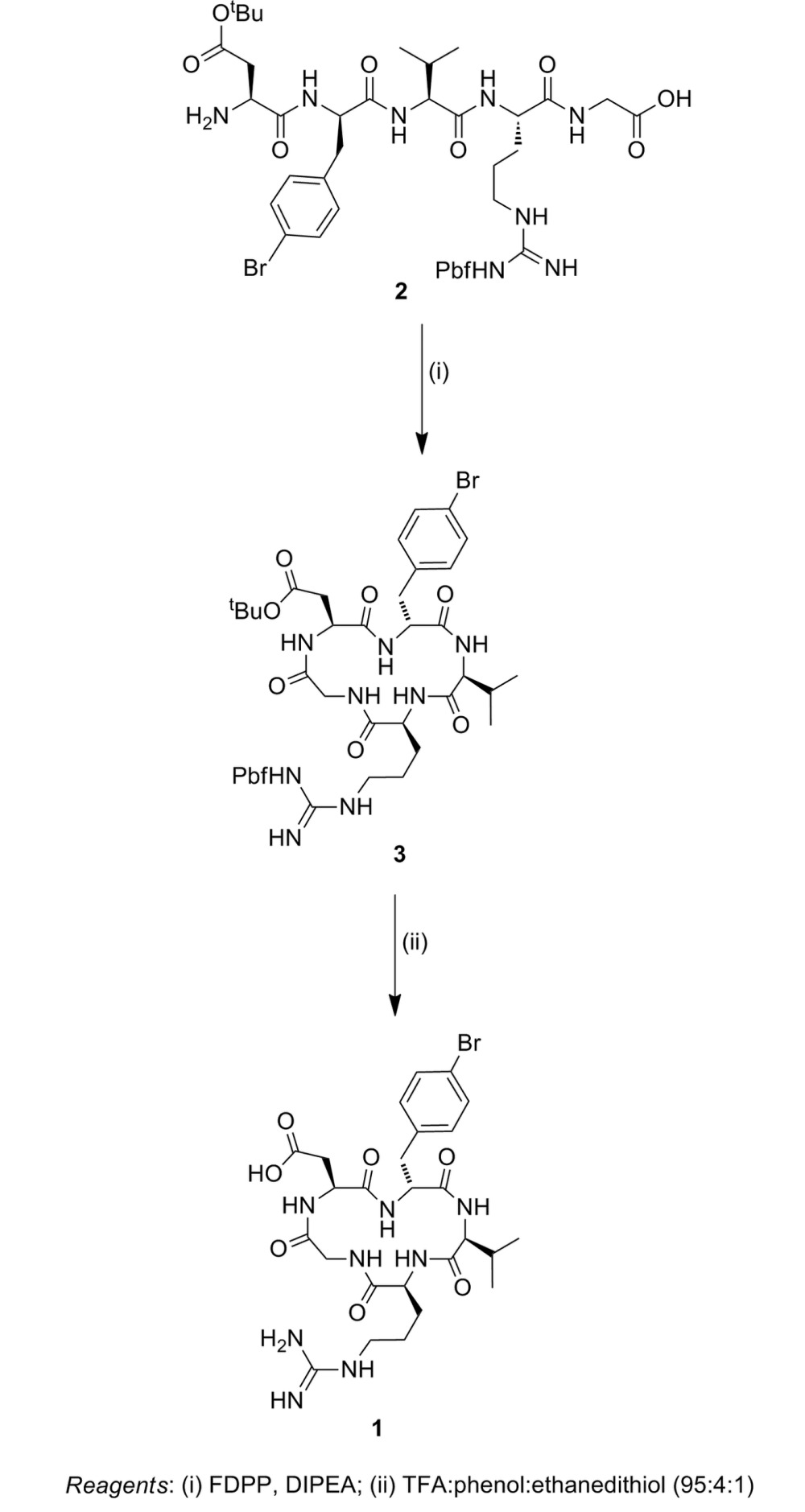


For treated B16 cells (Table 1 ▶, Fig. 2 ▶), a significant twofold increase in content of Br (*p* = 0.01) was observed when compared with control B16 cells (Fig. 3 ▶). A decrease in both Cl and K content (*p* = 0.01 and 0.02, respectively) was also observed (Table 1 ▶, Fig. 2 ▶) in comparison with B16 control cells. For the treated A549 cells (24 h incubation with 30 µ*M* of **1**) there was a significant increase observed in the content of K (*p* = 0.01) and a more than fourfold increase in Br content (*p* = 0.01) (Table 1 ▶, Fig. 4 ▶) when compared with A549 control cells (Fig. 5 ▶). No other statistically significant differences in intracellular elemental content were observed in either cell line.

Bromine was not found to be particularly localized in treated cells from either cell line; rather, bromine was found evenly distributed throughout the cells including the nucleus. We note, however, that given such an even distribution in combination with the low total Br density it is difficult to either demonstrate or disprove nuclear penetration of **1**. We also note that, along with the total levels of endogenous elements being predominantly undisturbed compared with the control, the cellular distributions of these elements were not notably modified as a result of treatment.

## 4. Discussion   

The Br elemental maps for both B16 and A549 treated cells (Figs. 2 ▶ and 4 ▶) show an even cellular distribution of Br. Many therapeutic drugs can be eliminated from their target cells through the action of lysosome (Woolf, 1999 ▶). However, if **1** was undergoing rapid degradation within lysosomes, the Br map would likely show a number of ‘hot spots’ corresponding to lysosomal accumulation, and only very low accumulation in other areas of the cell. This process was not observed; it is possible that the incubation time (24 h) and subsequent fixation procedure may result in lysosome accumulation events being missed.

The increase in Br content for treated B16 and A549 cells provides strong evidence that cyclic RGD peptide **1** accumulates within cells and is consistent with an interaction of the peptide with cell surface integrins such as α_v_β_3_, although we are unable to prove such an interaction using XRF. A number of studies have shown that covalent binding of RGD peptides and related peptidic motifs to drug molecules results in enhanced drug uptake, cellular accumulation and improvements in tumor-targeting (Ernst *et al.*, 2006 ▶; Bloch *et al.*, 2006 ▶; Ye *et al.*, 2006 ▶). Ernst *et al.* (2006 ▶) reported successful baculovirus transduction of mammalian cells as a result of an RGD-containing peptide, Xiong and co-workers have achieved enhanced intracellular drug delivery with RGD-labelled micelles (Xiong *et al.*, 2007 ▶), and cellular accumulation of cryptophane has been shown to increase when a peptide with a repeated RGD motif was conjugated to the molecule (Seward *et al.*, 2008 ▶). The mechanisms of integrin mediated cellular uptake have yet to be elucidated.

K is the most abundant intracellular ion in mammalian cells (Alberts *et al.*, 1994 ▶), and is found at high levels in both control and treated cells. Previous work has demonstrated that cell surface integrins can regulate the activity of K^+^ channels when mediated by endogenous ligands; for example, the binding of fibronectin to cell surface integrins has been shown to promote activation of hERG K^+^ channels (Hofmann *et al.*, 2001 ▶), laminin-binding has been demonstrated to promote inwardly rectifying K^+^ currents (Arcangeli *et al.*, 1996 ▶; Artym & Petty, 2002 ▶), and voltage-gated K^+^ channels such as K_v_1.3 have also been linked to integrin activation (Lewis & Cahalan, 1995 ▶; Artym & Petty, 2002 ▶). Upon treatment with **1**, A549 cells experienced a threefold increase in intracellular K content when compared with control. Conversely, in B16 cells, the K levels dropped significantly (Table 1 ▶, 67% decrease, *p* = 0.01) upon administration of **1**. This suggests that, while **1** does indeed bind to cell surface integrins on both A549 and B16 cells, the downstream regulation of K^+^ channels caused by the binding of **1** to integrin is substantially different between cell types (Arcangeli & Becchetti, 2006 ▶).

Possible cellular changes associated with apoptosis were observed in both **1** treated cell lines. Apoptosis consists of three stages: the initiation phase, the effector stage and the final degradation phase (Dallaporta *et al.*, 1998 ▶), and each phase is characterized by specific physical, biochemical and ionic changes. For the A549 cells, a significant decrease in cell size/volume was observed (Table 1 ▶, *p* = 0.01). During apoptosis, cell shrinkage occurs in its final stages, and it is driven by an earlier efflux of K^+^, Cl^−^ and other ions which draws water out of the cell through the osmotic gradient (Kerr *et al.*, 1972 ▶; Maeno *et al.*, 2000 ▶). However, the cell size decrease of approximately 20% observed in A549 cells treated with **1** is not large enough to confirm apoptosis, which typically reduce in size by more than 50% (Fernández-Segura *et al.*, 1999 ▶). Furthermore, the observed intracellular elemental changes indicative of apoptosis [including an increase in Ca^2+^ content and decrease in K^+^ and Cl^−^ ions (Fernández-Segura *et al.*, 1999 ▶)] were not evident.

The ability of all cells to maintain their volume during an osmotic challenge is dependent on the regulated movement of salt and water across the plasma membrane. An increase in the external concentration of ions can draw water out of cells, resulting in cell shrinkage (Alberts *et al.*, 1994 ▶). However, both the control cells and treated cells were subjected to the same conditions during culture, fixation and washing stages, and so this is unlikely to be the cause. At this juncture the underlying factors responsible for the reduction in size of the treated A549 cells compared with the control cells are yet to be determined.

In addition to the decrease in K content, a significant efflux of Cl (Table 1 ▶, 25% reduction, *p* = 0.01) was observed in B16 cells treated with **1**. Previous studies investigating cellular changes during the progression of apoptosis have shown that, despite the complexity of the process, changes in ionic content have a pivotal role (Fernández-Segura *et al.*, 1999 ▶; Yu & Choi, 2000 ▶). Concurrent K^+^ and Cl^−^ efflux (which is necessary to maintain electroneutrality) begins in the effector stage, prior to other indicators of apoptosis, such as Ca^2+^ increase and cell shrinkage, which occur in the final degradation stage (Fernández-Segura *et al.*, 1999 ▶; Yu & Choi, 2000 ▶; Dallaporta *et al.*, 1998 ▶). However, no significant decrease in cell size (*p* = 0.939) or increase in Ca content (*p* = 0.480) was noted. This suggests that the cells may be in the early stages of apoptosis, but not yet in the degradation phase. For the B16 cells, the higher concentration of **1** (75 µ*M*) administered compared with that used with the A549 cells (30 µ*M*), may be triggering a cytotoxic effect not seen in the A549 cells. Alternatively, the B16 murine melanoma cells may be more sensitive to the action of the RGD peptide. Further research is needed to elucidate the reasons for the observed differences in response between the B16 and A549 cells.

## 5. Conclusion   

This study represents the first example of synchrotron XRF imaging in cyclic peptide research and it complements those studies involving radiolabels and optical probes. This study demonstrates the first quantitative analysis of a heavy-atom labelled cyclic RGD peptide being incorporated into A549 and B16 tumor cells. The effect of **1** on the biodistribution of key endogenous elements at a sub-cellular resolution is also reported. The binding of cyclic RGD peptide **1** to integrins appears to mediate the cell specific efflux/influx of ions (K^+^ and Cl^−^); however, further work is needed to confirm the exact mechanism of action and their possible role in the disruption of tumor angiogenesis.

## Supplementary Material

Supporting information file. DOI: 10.1107/S0909049513001647/hf5218sup1.pdf

## Figures and Tables

**Table 1:** Mean elemental area densities (µg cm^−2^) and cell area (µm^2^) of cultured A549 control cells/A549 cells and B16 control cells/B16 cells treated with **1** The values are presented as an average of five technical replicates (*i.e.* data collected from five distinct single cells grown and treated within one culture well) with the standard deviation in the last decimal place provided in brackets.

	A549		B16	
	Control cell	Treated cell	Control cell	Treated cell
P	0.4 (2)	0.5 (2)	0.6 (1)	0.4 (3)
S	0.5 (2)	0.5 (2)	0.6 (1)	0.4 (3)
Cl	0.72 (4)	0.7 (1)	0.89 (4)	0.68 (4)†
K	0.03 (2)	0.09 (3)†	0.12 (1)	0.038 (4)†
Ca	0.12 (2)	0.08 (4)	0.13 (2)	0.12 (4)
Fe	0.012 (6)	0.012 (5)	0.0033 (6)	0.005 (1)
Cu	0.013 (3)	0.020 (7)	0.11 (9)	0.06 (4)
Zn	0.04 (1)	0.026 (9)	0.040 (9)	0.020 (2)
Br	4 (1) × 10^−4^	9 (2) × 10^−4^ †	2.9 (9) × 10^−4^	0.0013 (7)†
Cell area	260 (34)	200 (18)	630 (275)	640 (368)

† *p* ≤ 0.05.

## Footnotes

1 Supplementary data for this paper are available from the IUCr electronic archives (Reference: HF5218). Services for accessing these data are described at the back of the journal.
