# Supplementary material for: Synchrotron X-ray fluorescence studies of a bromine-labelled cyclic RGD peptide interacting with individual tumor cells
Source: J Synchrotron Radiat. 2013 Feb 7;20(Pt 2):226–33. doi: 10.1107/S0909049513001647 (PMC3943546; doi:10.1107/S0909049513001647)
Supplement: Supplementary file 1 [file s-20-00226-sup1.pdf]

Supplementary Information

Synchrotron XRF Studies of a Bromine-Labelled Cyclic RGD Peptide Interacting with Individual Tumor Cells

Erin J. Sheridan, Christopher J. D. Austin, Jade B. Aitken, Katrina A. Jolliffe, Louis M. Rendina*

School of Chemistry, The University of Sydney, Sydney, NSW 2006, Australia.

Stefan Vogt,

X-ray Science Division, Argonne National Laboratory, Argonne, Illinois 60439, USA.

Hugh H. Harris

School of Chemistry and Physics, The University of Adelaide, Adelaide, 5005, Australia.

Table S1. Elemental densities of each B16 cell treated with **1** in $\mu g \cdot cm^{-2}$.

Element	Elemental content of cell					Mean	Standard deviation
	A	B	C	D	E		
**P**	0.560	0.276	0.217	0.960	0.100	0.42	0.345
**S**	0.419	0.225	0.204	0.968	0.0882	0.38	0.349
**Cl**	0.676	0.691	0.698	0.622	0.722	0.68	0.037
**K**	0.0417	0.0196	0.0160	0.109	0.0046	0.038	0.0417
**Ca**	0.144	0.148	0.0842	0.147	0.0655	0.12	0.038
**Fe**	0.0057	0.0050	0.0038	0.0070	0.0036	0.0050	0.001
**Cu**	0.0840	0.0468	0.0323	0.105	0.0213	0.058	0.035
**Zn**	0.0262	0.0090	0.0105	0.0491	0.00405	0.020	0.0184
**Br**	1.44×10^{-3}	1.05×10^{-3}	7.36×10^{-4}	2.40×10^{-3}	6.24×10^{-4}	1.3×10^{-3}	7.2×10^{-4}

Table S2. Area of each B16 cell treated with **1** in μm^2.

	Elemental content of cell					Mean	Standard deviation
	A	B	C	D	E		
Cell area	295	415	439	940	1130	640	368

Table S3. Elemental densities of each B16 control cell in $\mu g \cdot cm^{-2}$.

**Element**	**Elemental content of cell**					**Mean**	**Standard deviation**
	**A**	**B**	**C**	**D**	**E**		
**P**	0.607	0.815	0.635	0.714	0.521	0.66	0.111
**S**	0.655	0.731	0.586	0.593	0.456	0.60	0.102
**Cl**	0.907	0.884	0.889	0.943	0.834	0.89	0.043
**K**	0.119	0.133	0.116	0.113	0.103	0.12	0.011
**Ca**	0.127	0.130	0.146	0.145	0.103	0.13	0.017
**Fe**	0.0033	0.0037	0.0032	0.0040	0.0023	0.0033	0.0006
**Cu**	0.128	0.0384	0.261	0.0735	0.0409	0.11	0.092
**Zn**	0.0434	0.0453	0.0244	0.0461	0.0382	0.040	0.0090
**Br**	2.92×10^{-4}	2.33×10^{-4}	4.32×10^{-4}	2.63×10^{-4}	2.18×10^{-4}	2.9×10^{-4}	8.6×10^{-5}

Table S4. Area of each B16 control cell in μm^2.

	**Elemental content of cell**					**Mean**	**Standard deviation**
	A	**B**	**C**	**D**	**E**		
Cell area	**489**	437	446	1087	679	630	275

Table S5. Elemental densities of each A549 cell treated with **1** in $\mu g \cdot cm^{-2}$.

Element	Elemental content of cell					Mean	Standard deviation
	A	B	C	D	E		
**P**	0.823	0.353	0.233	0.552	0.479	0.49	0.22
**S**	0.889	0.432	0.289	0.650	0.486	0.55	0.23
**Cl**	0.729	0.829	0.530	0.775	0.885	0.75	0.14
**K**	0.151	0.0653	0.0631	0.0973	0.0899	0.093	0.035
**Ca**	0.137	0.0477	0.0520	0.0677	0.0749	0.076	0.036
**Fe**	0.0192	0.00863	0.00831	0.0121	0.00925	0.012	0.0046
**Cu**	0.0302	0.0155	0.0158	0.236	0.0161	0.020	0.0065
**Zn**	0.0416	0.0191	0.0217	0.0263	0.0208	0.026	0.0092
**Br**	1.25×10^{-3}	6.92×10^{-4}	9.83×10^{-4}	8.16×10^{-4}	1.03×10^{-3}	9.6×10^{-4}	2.3×10^{-4}

Table S6. Area of each A549 cell treated with **1** in μm^2.

	Elemental content of cell					Mean	Standard deviation
	A	B	C	D	E		
Cell area	217.2	183.5	219.5	Excluded*	194.0	200	17.7

* Unable to accurately determine size due to cell leakage.

Table S7. Elemental densities of each A549 control cell in μg·cm^{-2}.

Element	Elemental content of cell					Mean	Standard deviation
	**A**	**B**	**C**	**D**	**E**		
**P**	0.485	0.700	0.287	0.319	0.378	0.43	0.17
**S**	0.515	0.760	0.270	0.383	0.367	0.46	0.19
**Cl**	0.655	0.718	0.747	0.729	0.736	0.72	0.036
**K**	0.0396	0.0563	0.0170	0.0194	0.0246	0.029	0.016
**Ca**	0.124	0.149	0.0967	0.134	0.0968	0.12	0.023
**Fe**	0.0160	0.0205	0.00761	0.0109	0.00703	0.012	0.0058
**Cu**	0.0138	0.0145	0.00882	0.0155	0.0134	0.013	0.0026
**Zn**	0.0491	0.0538	0.0244	0.0311	0.03542	0.039	0.012
**Br**	4.60×10^{-4}	5.52×10^{-4}	3.25×10^{-4}	3.56×10^{-4}	3.83×10^{-4}	4.2×10^{-4}	1.1×10^{-4}

Table S8. Area of each A549 control cell in μm^2.

	Elemental content of cell					Mean	Standard deviation
	A	**B**	**C**	**D**	**E**		
Cell area	260.8	276.5	241.8	215.3	307.5	260	34.9

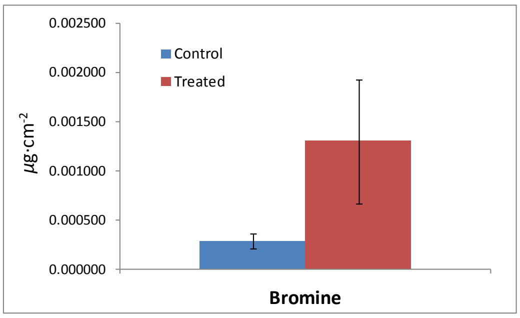

(i)

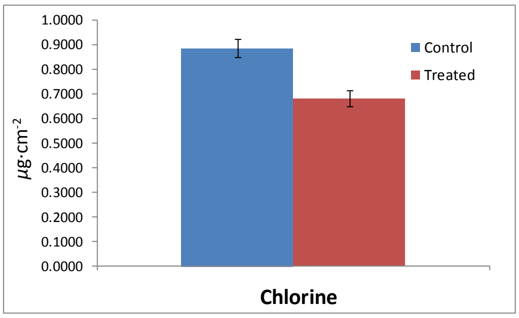

(ii)

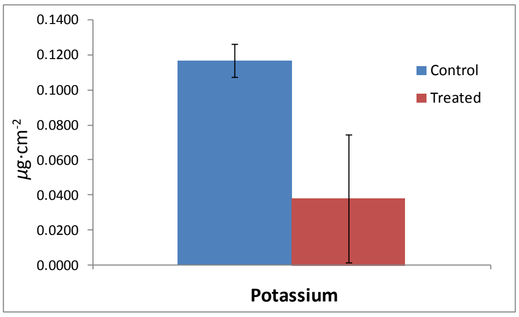

(iii)

Figure S1. Mean intracellular elemental content of treated vs. control B16 cells: (i) Br (p = 0.039), (ii) Cl (p = 0.00004), (iii) K (p = 0.012)

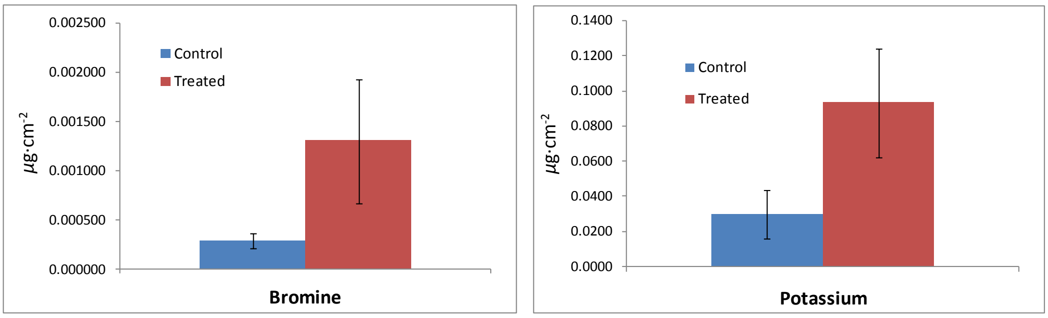

(i) (ii)

Figure S2. Mean intracellular elemental content of treated *vs*. control A549 cells:

(i) Br (p = 0.0028), (ii) K (p = 0.0119)

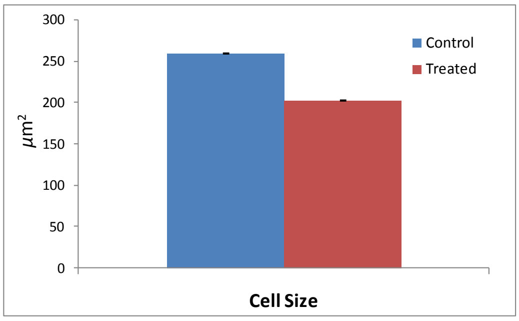

Figure S3. Mean cell size (μm^2) of treated vs. control A549 cells.

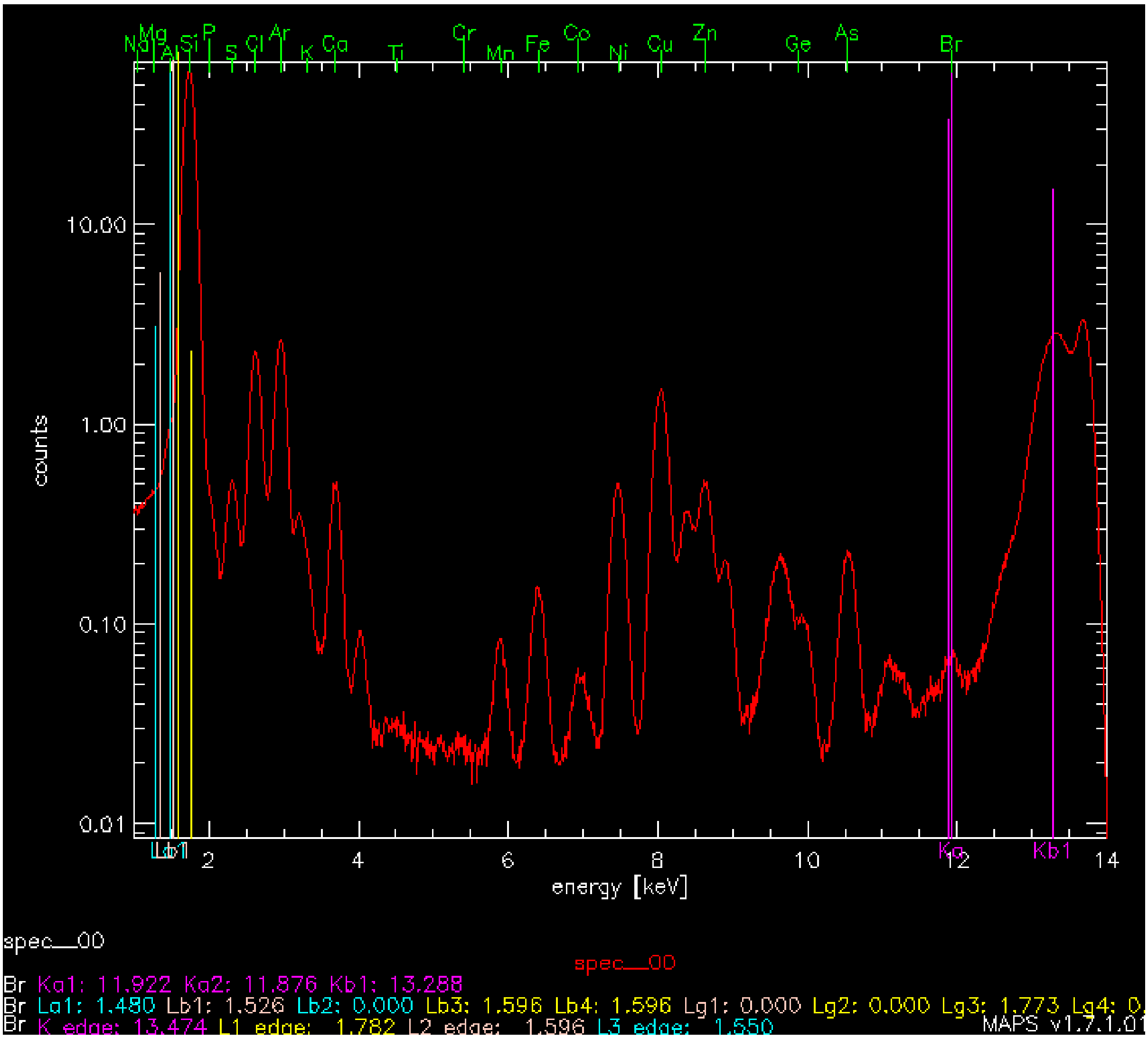

Figure S4. μ-XRF spectrum integrated across the region of interest of a single A549 cell treated with **1** for 24 h (cell from Figure 4). The Kα peak is at ~ 12 keV.

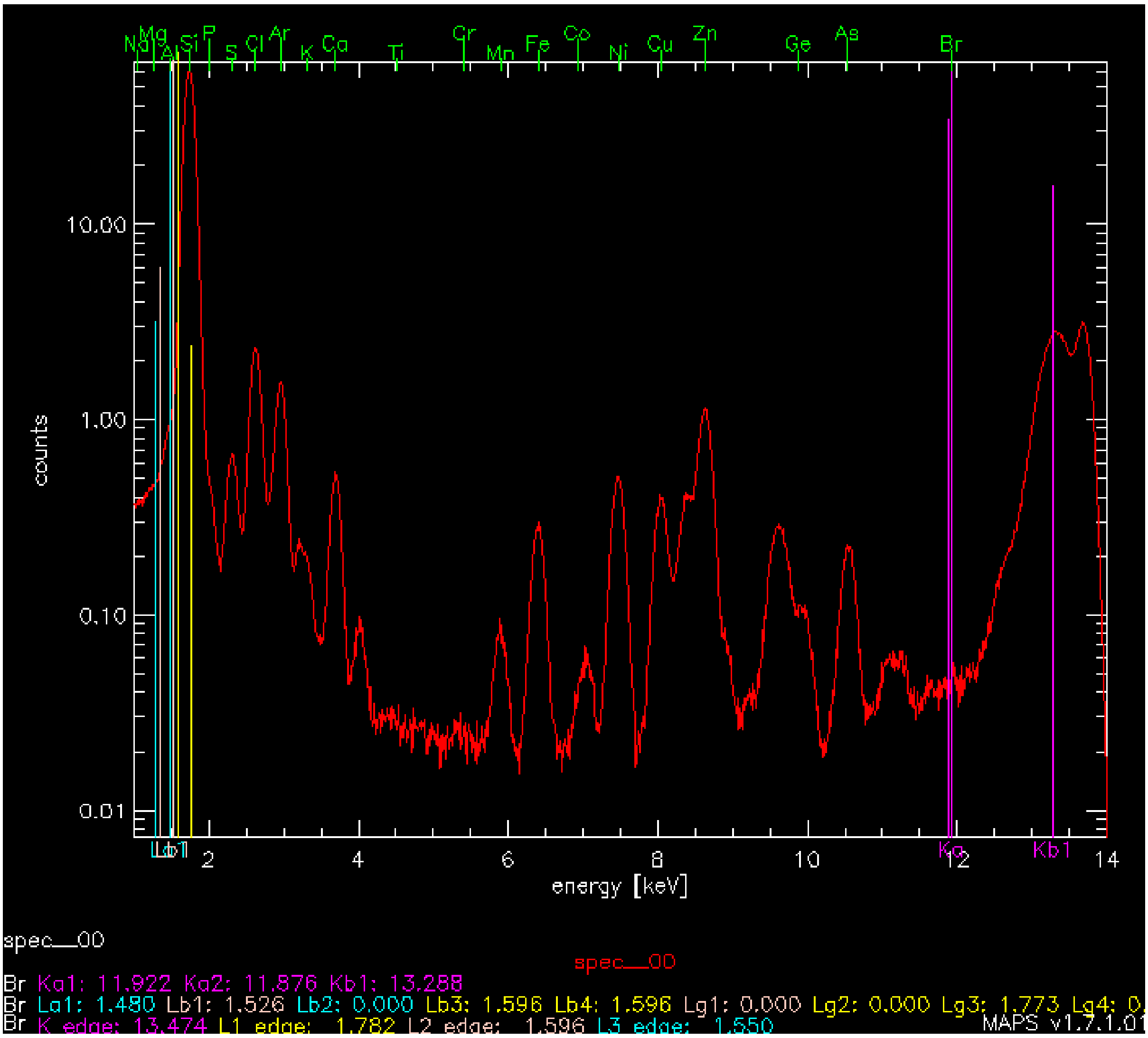

Figure S5. μ-XRF spectrum integrated across the region of interest of a single untreated A549 cell (cell from Figure 5). The Kα peak is absent from the region ~ 12 keV.
